# Celiac Disease Predisposition and Genital Tract Microbiota in Women Affected by Recurrent Pregnancy Loss

**DOI:** 10.3390/nu15010221

**Published:** 2023-01-01

**Authors:** Luca Masucci, Silvia D’Ippolito, Flavio De Maio, Gianluca Quaranta, Roberta Mazzarella, Delia Mercedes Bianco, Roberta Castellani, Annalisa Inversetti, Maurizio Sanguinetti, Antonio Gasbarrini, Giovanni Scambia, Nicoletta Di Simone

**Affiliations:** 1 Dipartimento di Scienze Biotecnologiche Di Base, Cliniche Intensivologiche e Perioperatorie, Università Cattolica del Sacro Cuore, 00168 Rome, Italy; 2 Dipartimento Scienze di Laboratorio e Infettivologiche, Fondazione Policlinico Universitario A. Gemelli Istituto di Ricovero e Cura a Carattere Scientifico (I.R.C.C.S.), 00168 Rome, Italy; 3 Dipartimento di Scienze della Salute della Donna, del Bambino e di Sanità Pubblica, Fondazione Policlinico Universitario A. Gemelli Istituto di Ricovero e Cura a Carattere Scientifico (I.R.C.C.S.), 00168 Rome, Italy; 4 Humanitas University Department of Biomedical Sciences, Humanitas University, via Rita Levi Montalcini 4, Pieve Emanuele, 20072 Milan, Italy; 5 IRCCS Humanitas Research Hospital, via Manzoni 56, Rozzano, 20089 Milan, Italy; 6 CEMAD Digestive Diseases Center, Fondazione Policlinico Universitario “A. Gemelli” IRCCS, Università Cattolica del Sacro Cuore, 00168 Rome, Italy; 7 Dipartimento di Medicina e Chirurgia Traslazionale, Università Cattolica del Sacro Cuore, 00168 Rome, Italy; 8 Dipartimento di Scienze della Vita e Sanità Pubblica, Università Cattolica del Sacro Cuore, 00168 Rome, Italy

**Keywords:** celiac disease predisposition, genital microbiota, recurrent pregnancy loss

## Abstract

The incidence of Idiopathic Recurrent Pregnancy Loss (RPL) is doubled in patients suffering from Celiac Disease (CD) compared to healthy populations. CD genetic components are HLA class II genes known as HLA-DQ2 and DQ8. Genetically susceptible women can remain asymptomatic even though they are exposed to a doubled risk of RPL compared to the general population. Furthermore, CD has been associated with microbiota alterations. The aim of this study is to evaluate endometrial and vaginal microbiota in HLA-DQ2/DQ8 positive and negative RPL patients compared to healthy pregnant women. Endometrial and vaginal microbiota of 3 subgroups were evaluated: 15 HLA-DQ2/DQ8 positive RPL women, 25 HLA DQ2/DQ8 negative RPL women (for a total of 40 RPL women) and 7 healthy fertile controls with previous uncomplicated pregnancies (all HLA-DQ2/DQ8 negative). The 2 RPL subgroups (HLA-DQ2/DQ8 positive and negative) showed a different endometrial and vaginal composition in the *Lactobacillacae* family compared to controls: *Lactobacillus acidophilus* was absent both in the vaginal and endometrial samples of RPL women, while *Lactobaciluus iners*, which can favor a less stable vaginal microbiota, was found only in RPL women (26.4% in HLA DQ2/DQ8 positive and 22.1% HLA DQ2/DQ8 negative) in both the vaginal and endometrial districts. In conclusion, both HLA DQ2/DQ8 positive-RPL and HLA DQ2/DQ8 negative-RPL women showed different endometrial and vaginal microbiota composition compared to healthy controls.

## 1. Introduction

According to the European Society of Human Reproduction and Embryology, recurrent pregnancy loss (RPL) is defined as the loss of two or more consecutive pregnancies before the 23rd week of gestation [1]. Several factors that have been linked to this condition—such as uterine anatomic anomalies, heritable and or acquired thrombophilia, uncontrolled diabetes mellitus, antiphospholipid antibody syndrome, infections, and environmental factors—but in about 40–50% of cases, etiology has not been identified [2].

This complication is a frustrating, overwhelming, and common disorder, affecting up to 5–7% of all women who are trying to conceive.

Over the last few years, researchers have shown the relationship between RPL and celiac disease (CD): in patients suffering from CD, the incidence of RPL is doubled compared to a healthy population [3,4]. As widely acknowledged, CD is a small bowel disorder characterized by mucosal inflammation, villous atrophy, and crypt hyperplasia caused by an abnormal immune/inflammatory response to dietary gluten in genetically susceptible individuals. The diagnosis of CD is established by the presence of intraepithelial lymphocytes with crypt hyperplasia alone or in conjunction with villous atrophy on small bowel biopsy in a patient with positive celiac serology.

CD genetic components are HLA class II genes known as HLA-DQ2 and DQ8 [5]. Approximately 25% of the general Caucasian population is HLA-DQ2/DQ8 positive but only 4% of them develop overt CD. CD patients are characterized by a broad symptomatology that includes different non-gastrointestinal symptoms such as associated autoimmune diseases, fatigue and growth retardation. At the same time, obstetrical complications, such as miscarriages, intrauterine growth restriction (IUGR), small for gestational age (SGA) babies, low birthweight (LBW) babies, or preterm birth can be related to CD [4]. CD heterogeneity in clinical presentation means that many atypical cases of CD go undiagnosed, potentially leading to a series of long-term complications [4].

Genetically susceptible women who are negative for anti-transglutaminase, -endomysium, and -gliadin antibodies do not undergo esophagogastroduodenoscopy (EGDS) and can remain asymptomatic for years. In our previous research we demonstrated that the prevalence of HLA-DQ2/DQ8 positivity in RPL women is around 53%, occurring almost twice as often as in the general population [5]. The mechanisms underlying this association have not yet been investigated. The effect of human leukocyte antigen genes (HLA) on intestinal microbiome has been already depicted: MHC complex and particularly HLA-DQ molecules are involved in immune activation leading to selection of the microbiota. Intestinal dendritic cells act as sentinels, sampling the mucosal surface for dietary or microbial antigens, activating immune cells that could favor tolerance or facilitate elimination of specific bacteria [6]. This has been already described in newborns [7]. Lessons learned from the intestinal microbiome suggested that the endometrial microbiota might modulate immune cells involved in implantation. This was also underlined by Benner et al. in 2018 [8]. Proving that a genetic predisposition to CD might be associated with a different genital tract microbiota in RPL women suggests the presence of a crosstalk between host genetic characteristics and tissue microbiota in the context of early pregnancy complications.

## 2. Materials and Methods

### 2.1. Patients

The study population included women with a history of idiopathic RPL who were negative for screening tests included in the RPL workup (anatomic, hormonal and/or metabolic, hematologic, autoimmune, genetic, thrombophilic testing) and healthy women with previous uncomplicated term pregnancies (control group). The inclusion criteria for both groups were as follows: Caucasian, age (≤44 years), healthy, regular ovulatory cycles (28–32 days), normal endocrine profile, normal serum levels of FSH (<10 mIU/mL), LH (<10 mIU/mL), and anti-müllerian hormone (AMH >2 ng/mL) on day 3 of the menstrual cycle, absence of abnormal ovarian and endometrial ultrasonographic features, no use of any contraceptive drugs or intrauterine device in the last six months; no vaginal infections; absence of ascertained intestinal organic diseases, including CD or inflammatory bowel diseases (Crohn’s disease, ulcerative colitis, diverticular disease, infectious colitis, ischemic colitis, microscopic colitis); no previous major abdominal surgeries; no previous bariatric surgery; no active malignancy of any type, or history of a malignancy; no untreated food intolerance such as ascertained lactose intolerance; no assumption of probiotics during the past three months before recruitment; no systematical/frequent use assumption of contact laxatives; ability to conform with protocol. After excluding the most common etiologies for RPL (uterine abnormalities, hormonal and metabolic disorders, severe male factor infertility, known clinical autoimmune diseases, thrombophilic conditions), RPL women with two or more spontaneous consecutive pregnancy losses (6–12 weeks of gestation) were clinically documented by ultrasonography and/or histopathological examination. All women did not receive any medications (such as anti-inflammatory, antibiotic or insulin-sensitizing drugs) in the past three months before being recruited for the study. All women were tested for HLA-DQ2DQ8 genotype on peripheral blood. Peripheral blood was collected in ethylene-diamino-tetra-acetic (EDTA) tubes from RPL and control women. HLA-DQ2/DQ8 analysis was performed after amplification of human DNA isolated from peripheral blood by real-time polymerase chain reaction following the manufacturer’s instructions (XeliGen RT, Eurospital SpA, Italy). Briefly, a PCR with sequence-specific primer tested for the following alleles: DQA1*01, DQA1*0201, DQA1*03, DQA1*05, DQA1*06, DQB1*02, DQB1*0301/03, DQB1*0301/04, DQB1*0302, DQB1*0305, and DQB1*04. The following DR alleles were typed in order to determine the presence of DQ/DR haplotypes: DRB1*03, DRB1*04, DRB1*07, DRB1*11. DQ2 positivity was defined as DQA1*05 in cohort with DQB2*02 (DQ2.5), or DQA1*0201 (DQ2.2)/DQA1*03 (DQ2.3) with B1*02. DQ8 positivity was defined as DQA1*03 with DQB1*0302.

Based on the presence of HLA-DQ2DQ8 genotype, RPL women were divided into two subgroups: HLA-DQ2/DQ8 positive-RPL and HLA-DQ2/DQ8 negative-RPL, respectively.

All women gave their informed consent to use, anonymously, their data for research purposes, and the protocol was approved by the ethics committee of A. Gemelli University Hospital, Università Cattolica del Sacro Cuore in Rome, Italy (ID 2363). All women were advised to avoid pregnancy in the month in which endometrial biopsy was performed.

### 2.2. Endometrial and Vaginal Samples

During the putative window of implantation (days 19–24), women underwent endometrial biopsies using a TAO Brush IUMC Endometrial Sampler (Cook Medical) to avoid vaginal and cervical contamination. The timing of the biopsy was based on the last menstrual period by measuring the follicle size with transvaginal ultrasound and was then confirmed by histologic evaluation.

Serum progesterone and beta subunit of human chorionic gonadotropin (β-hCG) levels were assessed on the day of the biopsy.

Cultural, histological, and functional examinations were carried out in all collected samples.

Endometrial samples were examined for the following infectious agents: *Chlamydia trachomatis*, *Ureaplasma* spp., *Mycoplasma* spp., *Neisseria gonorrhea*, *Yeasts*; human papillomavirus (HPV), Gram-negative and Gram-positive bacteria.

Women with evidence of chronic endometritis (CE) at hysteroscopy were not included in the study. The collected samples were split into two parts: one was put in 10% buffered formalin for paraffin embedding while the other one was preserved at −80 °C. Histologic dating of the endometrium was achieved according to standard criteria by a single investigator who was blinded to clinical outcomes. Histologic diagnosis of CE was based on the criteria described above [9,10].

We analyzed the following characteristics: superficial stroma edema, increased stroma density, and stromal inflammatory infiltrate dominated by lymphocytes and plasma cells.

In parallel, women underwent vaginal sample collection using a Copan swab (n. 490CE.AM). Similarly, the collected samples were analyzed for the following infectious agents: *Chlamydia trachomatis*, *Ureaplasma* spp., *Mycoplasma* spp., *Neisseria gonorrhea*, *Yeasts*, human papillomavirus (HPV), Gram-negative and Gram-positive bacteria.

In case of endometrial or vaginal infection, specific antibiotic therapy was prescribed, and patients were excluded from the study.

In the control group, no pathogens were identified in the vaginal and in endometrial samples.

### 2.3. Statistical Analysis

The results were expressed as mean ± standard deviation (SD) or percentage (%) of total. The data were analyzed using one-way analysis of variance (ANOVA) followed by a post-hoc test (Bonferroni test). Statistical significance was determined at *p* < 0.05.

### 2.4. Sequencing Procedures

Sample manipulation, DNA extraction and library preparation were performed.

Endometrial biopsies and vaginal swabs were stored at −80 °C until processing. Total DNA extraction was performed in a strictly controlled, separate and sterile workplace.

### 2.5. Extraction Procedures: Acid Nucleic Extraction from the Endometrium and Vaginal Swabs

Acid nucleic extraction from endometrial tissue samples and vaginal swabs was performed using QIAamp DNA Mini Kit (QIAGEN, Hilden, Germany). Endometrial biopsies (≤25 mg) were cut into small pieces and placed in a 1.5 mL microcentrifuge tube. Subsequently, 180 µL of Buffer ATL and 20 µL Proteinase K were added and incubated at 56 °C until complete lysis (1–3 h or overnight). The mixture was placed in a mini spin column.

Afterward, a series of washing and spinning processes were performed to obtain purified DNA. The same protocol, excluding tissue cutting, was used for vaginal swabs.

The quality and concentration of the extracted DNA were assessed for each sample by agarose gel electrophoresis and Qubit 4.0 fluorometer with ds DNA High sensitivity assay (Life Technologies). For each sample, the V3-V4 hypervariable regions of the 16S rRNA gene were amplified by using the following primers: V3_Next_For: 5′–TCGTCGGCAGCGTCAGATGTGTATAAGAGACAGCCTACGGGNGGCWGCAG–3′ and V4_Next_Rev: 5′–TCTCGTGGGCTCGGAGATGTGTATAAGAGACAGGACTACHVGGGTATCTAATCC–3′ [11].

The obtained amplicons were purified by using Agencourt AMPure XP beads (Beckman Coulter) and then barcoded with Nextera XT index (Illumina). Each indexed amplicon was equimolarly diluted, and the final pool was adequately prepared for the 2 × 300 paired ends sequencing process on the Illumina MiSeq instrument. To increase degree of base diversity, the internal control PhiX v3 (Illumina) was added to the library [11].

### 2.6. Bioinformatics Analyses

Raw sequencing data were demultiplexed to obtain FastQ sequences for each single sample and then analyzed by using Qiime2 [12]. Briefly, FastQ sequences were trimmed by Illumina adapters and not biological primer sequences and quality filtered. Amplicon Sequence Variants (ASVs) and chimera removal were obtained using the DADA2 algorithm [13]. Final taxonomic annotation was performed using a pre-fitted sklearn-based taxonomy classifier and SILVA 132 database [14]. Final data were pre-processed by removing mitochondrial sequences. Statistical analysis of microbiota diversity was performed in R studio (https://www.rstudio.com/ (accessed on 1 March 2022); version 4.0.2) using *phyloseq* package [15]. R software and R Studio were used to evaluate microbial community differences by measuring alpha and beta diversity after reading depth rarefaction. Shannon’s index and Pielou’s evenness index were used to evaluate microbial diversity and equitability among the samples (alpha diversity). Significance was assessed by the Mann Whitney U test. Beta diversity, which integrates phylogenetic relationships of bacteria, was calculated by weighted UniFrac and distance matrix represented as non-metric multidimensional scaling (NMDS) [16]. Significance was investigated by using Permutational multivariate analysis of variance (PERMANOVA). Relative abundances at Phylum and Genus level were reported and statistical significance was assessed by Mann–Whitney U test. Based on ASVs intrinsic characteristics and following previously used experimental setting [17,18,19,20], *Lactobacillacae* family was selected, and the relative abundance of *Lactobacillus* species was investigated. ASVs annotated as Lactobacillus species were additionally compared to the NCBI database to corroborate the previously obtained identification.

### 2.7. Sequencing Data of 16 S rRNA

In this study, we compared endometrial and vaginal microbiota of women with RPL and HLA-DQ2/DQ8 haplotypes, the main genetic predisposing factors for CD development, and healthy women (control group). Specimens of low quality or whole library size were excluded from the analysis.

Analysis of the endometrial samples resulted in 3,132,667 [median: 65637] reads grouped into 11,438 ASVs. A total of 3,044,501 [median: 65384] reads grouped into 395 ASVs remained after filtering out singletons and taxa representing fewer than 0.005% of all sequences. Conversely, 4,301,086 [median: 93076] reads were counted from vaginal samples grouped into 11,438 ASVs, of which 4,249,127 [median: 92163] reads clustered into 278 ASVs were recovered after filtering, as previously described. To compute alpha and beta diversity indices, reads of the endometrial and vaginal samples were normalized to 24,000 and 30,000, respectively.

## 3. Results

### 3.1. Patients

A total of 52 women with RPL and 8 control women were recruited over a period of 14 months (from July 2019 to September 2020) at the Recurrent Pregnancy loss Outpatient Clinic, Department of Obstetrics and Gynecology, Fondazione Policlinico Gemelli, IRCCS, Rome, Italy.

Women with evidence of chronic endometritis at hysteroscopy were not included in the study (n = 7 RPL). Furthermore, in case of endometrial or vaginal infection, specific antibiotic therapy was prescribed, and patients were excluded from the study (n = 5 RPL). At the analysis 16S rRNA gene sequencing data, specimens with low quality or whole library size were excluded from the analysis (n = 1 control). Finally, 40 RPL women were included in the analysis. The mean age of RPL women (n = 40) was 36.5 years (minimum 26 years, maximum 44 years, SD = 3.7). The mean age of the control group (n = 7) was 34.6 years (minimum 32 years, maximum 38 years, SD = 2.1). The number of smokers was lower among the control patients (0%) than the RPL women (22%). All major clinical characteristics of the study women were reported in Table 1. 

In the RPL group, all women had ≥2 miscarriages (100%). Regarding the number of deliveries at term (38–41 weeks), 25 RPL women (62.5%) had no deliveries, 11 RPL women (27.5%) had one delivery and 4 RPL women (10%) had more than one delivery; in the control group, women had 2 or more deliveries (100%).

Healthy fertile women with previous uncomplicated pregnancies represented the control group.

In agreement with a previous study, we observed a higher incidence of genetic predisposition to CD in idiopathic RPL women in comparison with the general population (52.6% vs. 23.6%), with a 3.6 times higher odds ratio of DQ2/DQ8 positivity in RPL women [5].

The study groups were divided as follows. In the RPL group, 15 women out of 40 (37.5%) were in the subgroup at risk of developing CD (HLA-DQ2/DQ8 positive-RPL), and 25 women out of 40 (62.5%) had no genetic risk of developing CD (HLA-DQ2/DQ8 negative-RPL).

All women in the control group (n = 7; 100%) had no risk of developing the CD.

### 3.2. Characteristics of the Endometrial Microbiota

**Figure 1:**
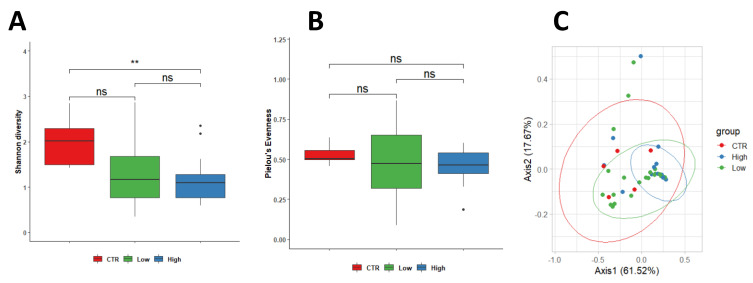
PERMANOVA statistical test showed a significant change between control and HLA-DQ2/DQ8 positive-RPL women in (*p* = 0.037, R^2^ = 0.109) in endometrial samples (Shannon diversity). No significant differences were observed between RPL women independent of the HLA-DQ2/DQ8 state (*p* = 0.900) (**A**,**B**). Differences in the microbial population between healthy control and women with CD predisposition were assessed using weighted Unifrac beta diversity. The obtained distance matrix was represented as PCoA, showing a clusterization among the three studied groups (**C**). Legend: CTR = control; Low = HLA-DQ2/DQ8 negative-RPL women; High = HLA-DQ2/DQ8 positive-RPL women. ** significantly different.

Microbial diversity was observed to be higher in the endometrium of control women (2.13 ± 0.58) compared to endometrium specimens from both subgroups of RPL (HLA-DQ2/DQ8 negative-RPL, 1.30 ± 0.70, *p* = 0.150; HLA-DQ2/DQ8 positive-RPL 1.15 ± 0.50; *p* = 0.006). In contrast, equitability appeared comparable among the three groups; values were 0.52 ± 0.06 in control women and 0.45 ± 0.23 (*p* = 0.590) in HLA-DQ2/DQ8 negative-RPL and 0.46 ± 0.10 (*p* = 0.220) in HLA-DQ2/DQ8 positive-RPL, respectively. No significant differences were observed between RPL women independently by the HLA-DQ2/DQ8 state (*p* = 0.900) (Figure 1A,B).

Our data indicated the existence of differences in the endometrial microbial populations of CTR women and RPL women in terms of bacterial diversity. Furthermore, these differences appeared to increase in HLA-DQ2/DQ8 positive-RPL (Figure 1A,B).

Differences in the microbial population between control and RPL women were assessed using weighted Unifrac beta diversity. The obtained distance matrix was represented as PCoA, showing a clusterization among the three studied groups (Figure 1C).

**Figure 2:**
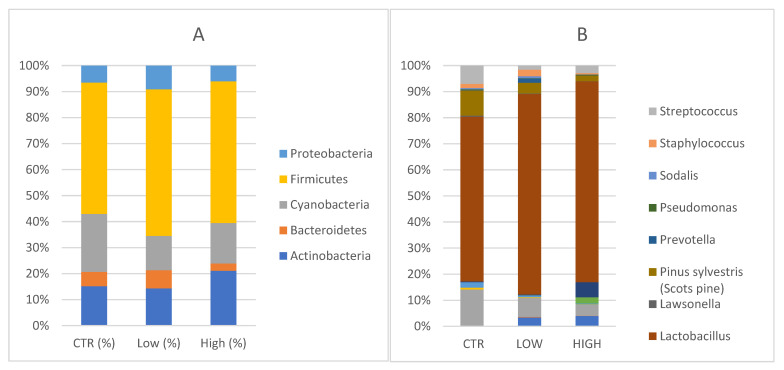
(**A**) Phylum level: Relative abundance detected in endometrial samples. CTR = control; Low = HLA- DQ2/DQ8 negative-RPL; High= HLA-DQ2/DQ8 positive-RPL. (**B**) Genus level: Relative abundances detected in endometrial samples. Legenda: CTR = control; Low = HLA-DQ2/DQ8 negative-RPL; High: HLA-DQ2/DQ8 positive-RPL.

Five main phyla *Actinobacteria*, *Bacteroidetes*, *Cyanobacteria*, *Firmicutes* and *Proteobacteria* were identified in endometrial specimens (Figure 2A).

Specifically, *Actinobacteria* were 16.4% [13.5, 7.8–22.4] in the control group in comparison with 17.7% [33.3, 2.0–15.7] (*p* = 0.262) in HLA-DQ2/DQ8 negative-RPL women and 29.3% [13.9, 5.6–51.2] (*p* = 0.808) in HLA-DQ2/DQ8 positive-RPL women. *Bacteroidetes* phyla constituted 6% [5.8, 3.8–8.1] in healthy subjects, 8.6% [6.3, 4.5–8.1] (*p* = 0.710) in HLA-DQ2/DQ8 negative-RPL, and 3.8% [3.0, 1.6–5.3] (*p* = 0.148) in HLA-DQ2/DQ8 positive-RPL women. *Cyanobacteria* accounted for 24.2% [22.7, 9.9–37.1] of the major phyla in endometrial samples of healthy women, 21.6% [16.8, 9.0–31.7] (*p* = 0.857) in the HLA-DQ2/DQ8 positive-RPL women, and 16.2% [5.6, 2.4–23.7] (*p* = 0.379) in the HLA-DQ2/DQ8 negative-RPL women. *Firmicutes* were not significantly increased in the HLA-DQ2/DQ8 positive-RPL group, showing an average of 75.6% [median: 95.9, 61.6–98.0] (*p* = 0.119) and HLA-DQ2/DQ8 negative-RPL group 69.4% [87.4, 43.4–94.8] (*p* = 0.262) compared to heathy subjects and presenting a relative abundance of 54.7% [53.4, 41.4–76.8]. *Proteobacteria* phyla constituted 7.1% [5.3, 4.4–10.0] in healthy subjects, 11.2% [8.1, 4.7–12.2] (*p* = 0.534) in HLA-DQ2/DQ8 negative-RPL women, and 8.4% [4.0, 1.8–7.4] (*p* = 0.319) in HLA-DQ2/DQ8 positive-RPL women.

Several genera were represented in endometrial samples (Figure 2B). *Lactobacillus* genus reached values of 48.4% [40.1, 27.0–76.5] in the control group, 66.2% [83.9, 43.5–94.8] in HLA-DQ2/DQ8 negative-RPL women, and 72.62% [95.9, 61.5–98.0] in HLA-DQ2/DQ8 positive-RPL women.

*Gardnerella* genus reached values of 0.18% in the control group, 0.26% in the HLA-DQ2/DQ8 negative-RPL subgroup and 5.45% in HLA-DQ2/DQ8 positive-RPL subgroup, showing a statistically significant difference (*p* = 0.025).

*Atopobium* spp. was detected in both RPL subgroups, showing values around 3% (2.92% in the HLA-DQ2/DQ8 negative-RPL subgroup and 3.75% in HLA-DQ2/DQ8 positive-RPL subgroup), but not in control group. These findings suggest slight changes in endometrium microbiota of CD predisposed women, even though the small sample size does not allow a specific microbial signature to be defined. 

### 3.3. Characteristics of the Vaginal Microbiota

**Figure 3:**
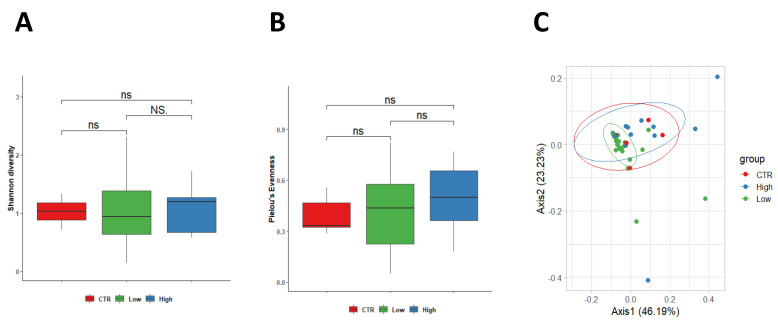
PERMANOVA statistical test confirmed a no significant change among the three groups (*p* = 0.224, R^2^ = 0.056) in vaginal samples. Legend: CTR = control; Low = Low-risk for CD; High: High-risk for CD.

When considering vaginal sample analysis, no differences were observed between control women and both subgroups of RPL women in terms of Shannon diversity index, which showed similar values among the three groups (healthy group 1.03 ± 0.23, HLA-DQ2/DQ8 negative 1.09 ± 0.63 and HLA-DQ22/DQ8 positive 1.08 ± 0.37) (Figure 3A). Although a positive trend was found in Pielou’s evenness in women HLA-DQ2/DQ8 RPL women, no significant difference was measured between the control group (0.392 ± 0.11) in comparison with HLA-DQ2/DQ8 negative-RPL women (0.49 ± 0.21, *p* = 0.560) and HLA-DQ2/DQ8 positive-RPL women (0.49 ± 0.18, *p* = 0.190). Furthermore, no differences were observed among the two RPL subgroups (*p* = 0.270) (Figure 3B). Similarly, weighted Unifrac beta diversity did not show any significant spatial clusterization among samples (*p* = 0.224, R^2^ = 0.056) (Figure 3C).

**Figure 4:**
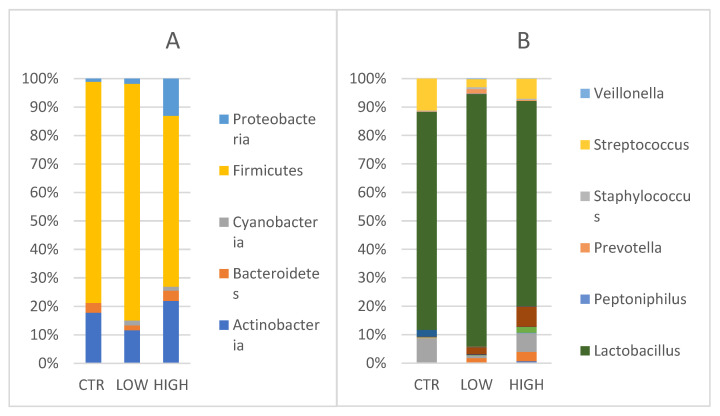
(**A**) Phylum level: Relative abundance detected in vaginal samples CTR = control; Low = HLA DQ2 DQ8 negative-RPL; High: HLA DQ2 DQ8 positive-RPL. (**B**) Genus level: Relative abundances detected in vaginal samples. Legenda: CTR = control; Low = HLA DQ2 DQ8 negative-RPL; High: HLA DQ2 DQ8 positive-RPL.

Vaginal Phyla composition was evaluated (Figure 4A). *Actinobacteria* showed values of 20.6% [24.4, 15.1–28.0] in control women, 12.77% [3.8, 2.7–18.6], respectively) in HLA-DQ2/DQ8 negative-RPL women and 29% in HLA-DQ2/DQ8 positive-RPL women [24.5, 8.9–46.7]. *Bacteroidetes* and *Cyanobacteria* were found only in CD predisposed vaginal samples. *Bacteroidetes* accounted a relative abundance of 4.8% [1.9, 1.1–6.6] and 4.0% [4.0], whereas the latter showed values of 1.9% [1.7, 1.4–2.3] and 1.8% [1.4, 1.2–2.2] in negative and positive groups, respectively.

*Firmicutes* were found in the control group (90.1%) [97.8, 84.0–99.4] (*p* = 0.378), in the HLA-DQ2/DQ8 negative-RPL women (91.3%) [96.9, 89.0–98.6], and in the HLA-DQ2/DQ8 positive-RPL women (79.6%) [89.8, 71.5–99.2] (*p* = 0.308). *Proteobacteria* were detected in all groups, showing a comparable value between control group (1.2%) and HLA-DQ2/DQ8 negative-RPL women (2%, respectively) with respect to HLA-DQ2/DQ8 positive-RPL women where relative abundance was of 17.3% [17.3, 10.9–23.7].

When considering genera, (Figure 4B) *Lactobacillus* genus was predominant in the three groups. *Lactobacillus* reached values of 76.2% [97.7, 71.0–99.3] in the control women, 87.4% [95.6, 81.8–98.3] in the HLA-DQ2/DQ8 negative RPL women and 71.5% [88.5, 55.0–99.1] in the HLA-DQ2/DQ8 positive RPL women, even though no statistically significant difference was observed among the groups.

Conversely to the *Lactobacillus* trend, *Streptococcus* genus reached values of 11.3% in the control group, 2.75 % in HLA-DQ2/DQ8 negative-RPL women, and 6.97% in HLA-DQ2/DQ8 positive-RPL women. As observed for endometrium samples, *Gardnerella* and *Atopobium* genera were mainly represented in RPL CD predisposed patients as reported (2.4% and 1.61% in HLA-DQ2/DQ8 negative-RPL women, 7% and 3.27% in HLA-DQ2/DQ8 positive-RPL women, respectively)(Figure 4B).

### 3.4. Differences in Lactobacillus Species

We selected *Lactobacillacaee* family and detected seven *Lactobacillus* species, considering the representation within the samples (≥1%): *L. acidophilus*, *L. crispatus*, *L. delbruekii*, *L. gallinarum*, *L. gasseri*, *L. iners*, and *L. jensenii*. Healthy control endometrial samples were mainly constituted by *L. acidophilus*, *L. crispatus*, and *L. gasseri*, which accounted for 18.32%, 47.58, and 22.8%, respectively. A similar pattern was found also in the healthy control vaginal sample: *L. acidophilus* (13.8%), *L. crispatus* (44.6%), and *L. gasseri* (22.8%). No difference was observed between healthy controls and HLA-DQ2/DQ8 positive-RPL women.

**Figure 5:**
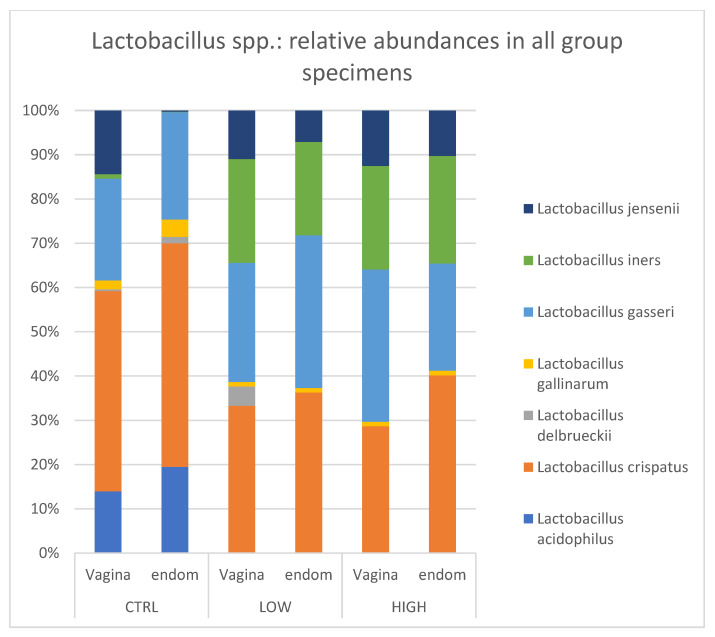
*Lactobacillus* spp.: relative abundances in all group specimens. Legend: CTR = control; Low = HLA DQ2 DQ8 negative-RPL; High: HLA DQ2 DQ8 positive-RPL.

Interestingly, in both subgroups of RPL women (HLA-DQ2/DQ8 positive and negative), *L. acidophilus* was not detected and *L. iners* was equally distributed in endometrial (ranging from 20.7% to 23.5%) and vaginal (ranging from 22.7 to 23.0%) samples. Conversely, a very low value of the *L. iners* relative abundance was detected in the control group (1% in vagina and 0.1% in endometrium) (Figure 5).

## 4. Discussion

Recurrent pregnancy loss (RPL) still represents a pregnancy complication with unknown etiology in about 40% of cases. This makes idiopathic RPL an incredibly stressful event for couples trying to conceive, thus emphasizing the need for more specific studies in this area. An increased risk of miscarriage has been associated with CD, with a relative risk (RR) of 1.39 [5]. Conversely, women with RPL have a nearly 6-fold increased risk of being affected by CD compared with the general population (Odds Ratio, OR 5.82, 95% CI 2.30–14.74). As we know, human leukocyte antigen (HLA) DQ2/DQ8 haplotypes represent the major determinants of genetic susceptibility to CD. They codify for the DQ2/DQ8 proteins, responsible, in celiac subjects, for the presentation of immunogenic gluten peptides to DQ2/DQ8-restricted CD4^+^ T cells [21,22,23]. Once activated, CD4^+^ T cells enhance a complex immune response with increased production of Interferon (IFN)-γ, Tumor Necrosis Factor (TNF)-α and autoantibodies, like anti-transglutaminase, -endomysium, and -gliadin antibodies. In agreement with our previous research, we observed a higher prevalence of HLA-DQ2/DQ8 positivity in women with idiopathic RPL compared to the general population. By trying to explain such observations, we may suggest that HLA-DQ2/DQ8 positivity, in the presence of exogenous stimuli, may favor an inflammatory condition with detrimental effects during the early stages of pregnancy. It has been demonstrated that microbiota alterations (dysbiosis) can, by interacting with the immune system, promote excessive inflammatory tissue reactions which disturb the local homeostasis [24,25,26]. We, therefore, investigated the endometrial and vaginal microbiota in RPL women in relation to their HLA-DQ2/DQ8 state. By comparing healthy fertile control women and RPL women with or without the HLA-DQ2/DQ8 mutated gene (HLA-DQ2/DQ8 positive-RPL and HLA-DQ2/DQ8 negative-RPL, respectively), we did not find substantial differences among the three subgroups when considering alpha bacterial diversity. In contrast, we observed an overlapping, but not statistically significant, distribution between healthy controls and HLA-DQ2/DQ8 positive compared to HLA-DQ2/DQ8 negative RPL women when considering beta diversity.

Vaginal and endometrial microbiota showed differences in relative abundances of the five major phyla *Actinobacteria*, *Bacteroidetes*, *Cyanobacteria*, *Firmicutes* and *Proteobacteria*. Specifically, *Cyanobacteria* were detected only in vaginal samples from CD predisposed women but were in endometrial samples of all study groups. *Cyanobacteria* are Gram-negative bacteria that produce three types of toxin: hepatotoxins, neurotoxins, and lipopolysaccharide endotoxins. Commonly, these bacteria cause acute gastroenteritis following the consumption of contaminated drinking water. Their potential effects on the female genital tract are currently unknown. *Bacteroidetes* and *Proteobacteria* appeared to be more abundant in the endometrium than in the vagina in each of the three groups, confirming previous descriptions about female genital microbiota [27].

In the endometrium, *Bacteroidetes* and *Proteobacteria* were equally distributed among the three groups, while *Firmicutes* were more prevalent in the HLA-DQ2/DQ8 negative subgroup (86.38%) compared to healthy women (≈67.5%) and the HLA-DQ2/DQ8 positive RPL subgroup (65.2%). In the vaginal samples, *Firmicutes* were the most represented, but they were lower in HLA-DQ2/DQ8 positive RPL women (≈80%) with respect to the HLA-DQ2/DQ8 negative RPL women (≈91%) and the control group (≈90%). *Bacteroidetes* were equally represented in vaginal samples of the RPL women subgroups but not in the control group. *Proteobacteria* were detected in all groups, but there was a significant increase of relative abundance in HLA-DQ2/DQ8 positive-RPL women compared to controls and the HLA-DQ2/DQ8 negative RPL subgroup.

Within the Firmicutes, the most prominent component is represented by *Lactobacillus* genus. In healthy conditions, females host a dominance of *Lactobacillus* bacteria which exert many protective functions connected to lactic acid production and to maintaining an acid vaginal pH [28]. Several studies have reported the association between low amount of *Lactobacillus* spp. and adverse reproductive outcomes, including infertility, preterm birth, and sexually transmitted diseases [29,30,31,32,33,34,35]. According to *Lactobacillus* species abundance, different vaginal microbiotas have been clustered into 5 specific community state types (CST). Particularly, CST I is dominated by *Lactobacillus crispatus*, CST II by *Lactobacillus gasseri*, CST III by *Lactobacillus iners*, and CST V by *Lactobacillus jensenii*; CST IV is defined as a low *Lactobacillus* spp. predominance and includes a diverse set of strict and facultative anaerobes. In our study groups, seven *Lactobacillus* spp. in the endometrial and vaginal samples have been detected: *L. acidophilus*, *L. crispatus*, *L. delbruekii*, *L. gallinarum*, *L. gasseri*, *L. iners* and *L. jensenii*. Regarding the endometrium, we found that (i) *L. acidophilus* (18.32%), *L. crispatus* (47.6%), and *L. gasseri* (22.8%) were more common in the healthy group; (ii) *L. iners*, *L. crispatus and L. gasseri* were equally distributed in HLA-DQ2/DQ8 negative and positive RPL women (≈24.4%, 31.3% and 27.8% respectively; (iii) *L. acidophilus* was not detected in either RPL subgroup as compared to the healthy control.

On the other hand, the vaginal microbiota showed typical *Lactobacillus* species distributions. *L. crispatus*, *L. gasseri* and L. *jensenii* were almost equally distributed among the three subgroups. *L. acidophilus* was highly present in healthy women (≈13%), but it was absent in both RPL subgroups. *L. iners* was found only in RPL women (≈26.4% and ≈22.1% in HLA-DQ2/DQ8 negative and HLA-DQ2/DQ8 positive RPL, respectively). These data demonstrated that control and RPL groups showed a different endometrial and vaginal composition mainly in the *Lactobacillacae* family. In particular, the increased *L.iners* expression found in both the vaginal and endometrial districts of RPL women might indirectly suggest a possible marker of increased risk of RPL. Differences in the abundance of Lactobacillus are important for pregnancy prognosis. It is known that healthy pregnancy is mainly characterized by a stable vaginal bacterial composition with *Lactobacillus* spp. dominance and low bacterial diversity: *L*. *gasseri* and *L. iners* are associated with pH values >4.0 and a less stable vaginal microbiota, so they in turn are more favorable to induce the development of bacterial vaginosis (BV), whereas *L. crispatus* is associated with pH values <4.0. In addition, vaginal dysbiosis can result in BV, which is associated with poor pregnancy outcome [31]. Thus, not all *Lactobacillus* spp. have a protective role.

This evidence, taken together and associated with the bacteria imbalance described above, can lead us to hypothesize a predisposition to altered vaginal environment of HLA-DQ2/DQ8 positive-RPL women.

The main finding of the present study is the observation of different bacterial population at the endometrial level in idiopathic RPL women, particularly in the *Lactobacillacae* species. According to our hypothesis, our findings suggested a possible interaction between the host genetic characteristics and microbiota, possibly leading to an inflammatory response that has been already suggested to create an unfavorable environment for early implantation [36,37,38]. Regarding idiopathic RPL, new frontiers of investigation are urgently needed. In this context, starting from a previous study by the same authors [3] on the association between HLA DQ2/DQ8 positivity and idiopathic RPL, we moved to investigate the relationship between HLA-DQ genes and the vaginal-endometrial microbial colonization process. The ultimate aim of this work will be to establish a classification of RPL women based on bacterial patterns, which would allow a personalized diagnosis based on the microbiota. In addition, these findings suggested that the normalization of dietary composition via food, probiotics, and lifestyles changes before conception and also during early pregnancy (“early life nutrition”) may impact the composition of endometrial and vaginal microbiota. Obviously, further studies, based on a larger sample size, are needed to better understand the endometrial microbiota and thereby allow new rational approaches for a diagnosis and a personalized medicine.

## Figures and Tables

**Table 1:** Characteristics of women included in the study. Results are expressed in average ± standard deviation (SD) or percentage (%) of the total, where deemed appropriate. CTR control women; RPL recurrent pregnancy loss; n: number, BMI body mass index, NS not significant, N/A not available.

Characteristics of Women	CTR Group (n = 7)	RPL Group (n = 40)	*p* Value
Age (years) at first visit ± SD	34.6 ± 2.1	36.5 ± 3.7	NS
BMI (Kg/m^2^) ± SD	20.63 ± 1.04	22.33 ± 2.84	NS
Cigarette smoking (%)	0 (0%)	7 (22%)	NS
Ethnicity			
Caucasian	7 (100%)	40 (100%)	
Number of miscarriages	0	3.26 ± 1.16	0.00001
(6–12 weeks)			
Women with ≥2 miscarriages (%)	0 (0%)	40 (100%)	
Number of deliveries at term (38–41 weeks)			0.0079
0 delivery	0 (0%)	25 (62.5%)	
1 delivery	1 (12.50%)	11 (27.5%)	
>1 delivery	6 (87.50%)	4 (10%)	
HLA DQ2/DQ8 state			
positive	0 (0%)	15 (37.5%)	
negative	0 (0%)	25 (62.5%)	

## Data Availability

Data are available upon request to the corresponding author.
